# Robotic transcranial magnetic stimulation in the treatment of depression: a pilot study

**DOI:** 10.1038/s41598-023-41044-1

**Published:** 2023-08-28

**Authors:** Hyunsoo Shin, Hyeonseok Jeong, Wooseok Ryu, Geunhu Lee, Jaeho Lee, Doyu Kim, In-Uk Song, Yong-An Chung, Sungon Lee

**Affiliations:** 1 Department of Electrical and Electronic Engineering, Hanyang University, Ansan, 15588 Republic of Korea; 2 Department of Radiology, Incheon St. Mary’s Hospital, College of Medicine, The Catholic University of Korea, Seoul, 21431 Republic of Korea; 3 Department of Neurology, Incheon St. Mary’s Hospital, College of Medicine, The Catholic University of Korea, Seoul, 21431 Republic of Korea; 4 Tesollo Inc., Gwangmyeong, 14353 Republic of Korea; 5 Department of Nuclear Medicine, Incheon St. Mary’s Hospital, College of Medicine, The Catholic University of Korea, Seoul, 21431 Republic of Korea; 6 Department of Robotics, Hanyang University, Ansan, 15588 Republic of Korea

**Keywords:** Electrical and electronic engineering, Biomedical engineering, Mechanical engineering

## Abstract

There has been an increasing demand for robotic coil positioning during repetitive transcranial magnetic stimulation (rTMS) treatment. Accurate coil positioning is crucial because rTMS generally targets specific brain regions for both research and clinical application with other reasons such as safety, consistency and reliability and individual variablity. Some previous studies have employed industrial robots or co-robots and showed they can more precisely stimulate the target cortical regions than traditional manual methods. In this study, we not only developed a custom-TMS robot for better TMS coil placement but also analyzed the therapeutic effects on depression. Treatment effects were evaluated by measuring regional cerebral blood flow (rCBF) using single-photon emission computed tomography and depression severity before and after rTMS for the two positioning methods. The rTMS preparation time with our robotic coil placement was reduced by 53% compared with that of the manual method. The position and orientation errors were also significantly reduced from 11.17 mm and 4.06° to 0.94 mm and 0.11°, respectively, confirming the superiority of robotic positioning. The results from clinical and neuroimaging assessments indicated comparable improvements in depression severity and rCBF in the left dorsolateral prefrontal cortex between the robotic and manual rTMS groups. A questionnaire was used to determine the patients’ feelings about the robotic system, including the safety and preparation time. A high safety score indicated good acceptability of robotic rTMS at the clinical site.

## Introduction

Transcranial magnetic stimulation (TMS) is a noninvasive treatment that uses magnetic fields to stimulate neurons in the brain. Depending on the purpose of the therapeutic application, the target of stimulation should be locally focused on a specific area of the brain. As a representative field of application, the treatment effect of depression through repetitive TMS (rTMS) has been proven in patients who fail to achieve a satisfactory response to antidepressants^1–7^. As a depression treatment session with rTMS takes approximately 20 min, the TMS coil is commonly held using a mechanical holder after positioning the target. To move the position and direction of the TMS coil to the stimulus point, the clinician adjusts the coil while checking the stimulus point through neuronavigation^8–10^ or places the coil according to the 10–20 system^11^.

For the rTMS treatment of depression, the left dorsolateral prefrontal cortex (DLPFC) is usually used as the target brain stimulation area, as functional neuroimaging studies have demonstrated left-dominant hypoperfusion and hypometabolism in this region in patients with depression^12,13^. Moreover, increases in regional cerebral blood flow (rCBF) and glucose metabolism are associated with improvements in depressive symptoms^14,15^. To find the left DLPFC, clinicians usually use a simple method called the 5 cm rule, which detects the motor cortex area and considers the stimulus point 5 cm ahead from that location, if they do not have neuronavigation^3,4^. However, as pre-operation before the treatment session, including the procedure to determine the stimulus point, is a difficult task, it requires a long preparation time, which leads to serious discomfort for the clinician and patient, particularly with depressive symptoms. Furthermore, because of the size and shape of the brain, the distance from the coil to the neurons, and the different anatomical structures for each patient, the neuronavigation method has been developed to provide precise spatial information to the clinician^9^.

Accurate positioning of a coil in TMS is important for several reasons. First of all, it is crucial because TMS targets specific brain regions. TMS is used to stimulate specific areas of the brain, and accurate positioning of the coil is necessary to target the desired brain region. Precise targeting is important for both research and clinical applications, as it allows for a more targeted and effective stimulation. Second, accurate coil positioning is also important for safety reasons. TMS can produce powerful magnetic fields that can potentially cause harm if not properly positioned. For example, incorrect placement of the coil can result in stimulation of the wrong part of the brain, which can cause unwanted side effects. Third, accurate coil positioning is crucial for obtaining consistent and reliable results in TMS studies. Even small variations in coil position can lead to differences in the effects of TMS stimulation. Researchers need to be able to replicate their findings across different experiments, and this requires consistent and precise coil positioning. Fourth, accurate positioning allows for the customization of TMS stimulation to each individual, maximizing the effectiveness of the treatment or research intervention. The position of the coil can also vary depending on the individual's head shape and anatomy. Last, the accuracy of coil positioning can also impact the optimization of TMS stimulation parameters, such as the intensity and frequency of the stimulation. Accurate positioning allows for precise targeting and adjustment of these parameters, which can improve the effectiveness of the stimulation and minimize side effects.

For these reasons, there have been studies on robotized TMS^16–23^and they have shown TMS using the positioning robots lead to improved procedure time and intraoperative accuracy compared with the traditional method. While it is important to validate system performance in the lab, it is even more important to validate performance and see how patients feel through testing in the clinical environment in which the system will be used. Automated positioning robotic system may make patients apprehensive about unexpected accidents, such as collisions with robots. Although studies on developing robotized TMS and the automatic detection of motor evoked potentials have been conducted^24,25^, studies comparing manual and robotic devices for patients are scarce.

**Figure 1:**
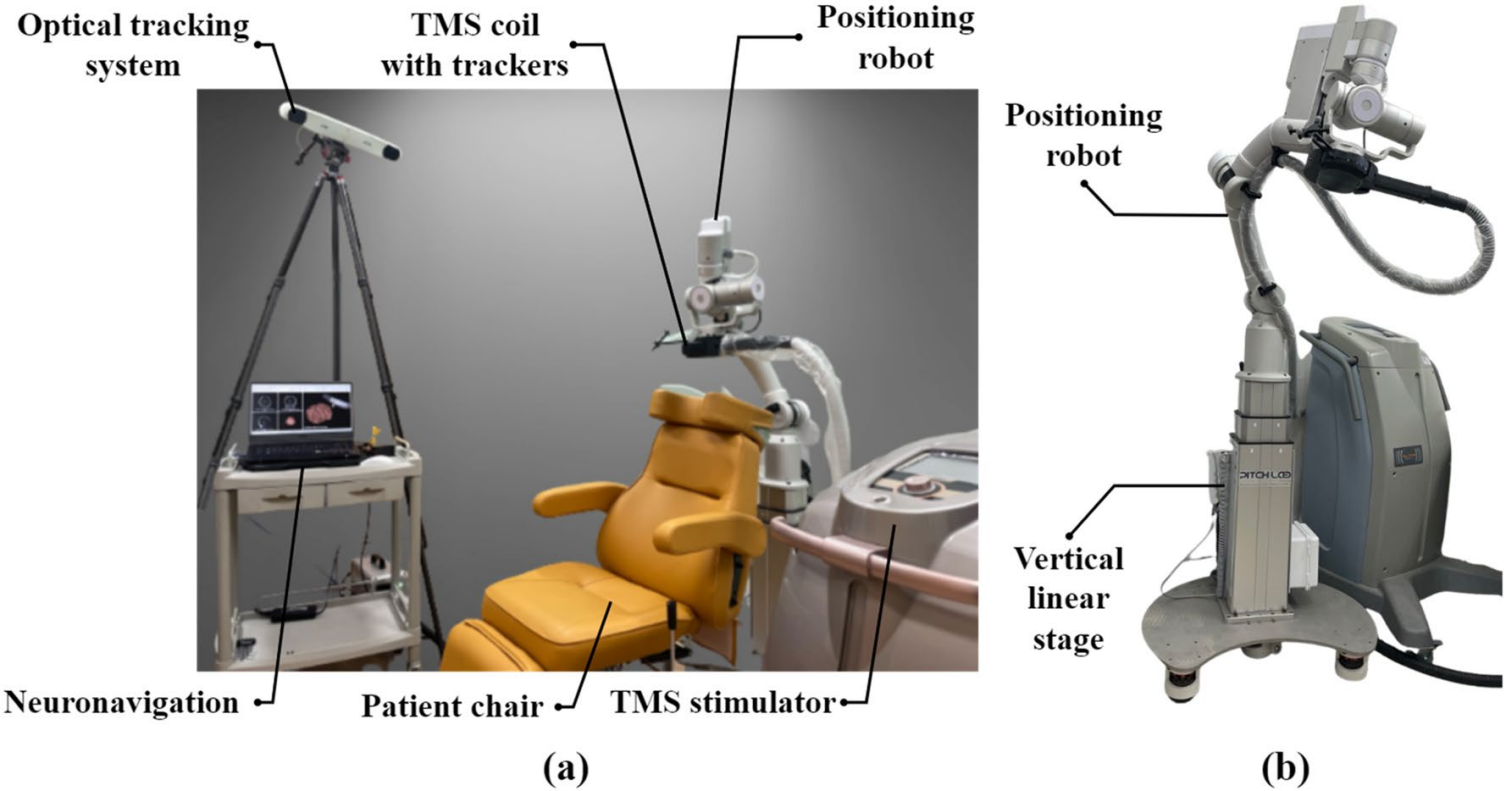
(**a**) Setup of the robotic repetitive transcranial magnetic stimulation (rTMS) in our treatment room, (**b**) positioning robot for automatic coil placement.

Therefore, one purpose of this study was to validate the accuracy and setup time of coil placement using robotic rTMS for depression treatment. Figure 1 and Table 1 show our setup of the treatment and explain the role of each system. We measured the relative distance between the patient’s head and the TMS coil using a 3D tracking system to verify the accuracy of the coil placement. As the relative movement of the patient’s head might affect the outcome during the treatment session, we also evaluated the error between the target cortical area and the focus of the coil. Our secondary purpose was to verify the changes in depression severity and rCBF after the robotic rTMS treatments using single-photon emission computed tomography (SPECT). Therefore, our study not only confirms the feasibility of the robotic rTMS method for the treatment of depression but also verifies the accuracy of the coil placement affecting the therapeutic effect.

**Table 1:** Description of the role of each system for automatic coil placement.

System	Role of each system	Specification
Optical tracking system	Pose estimation of the TMS coil and human head	Volume accuracy: 0.3 mm
Positioning robot	Placing the TMS coil precisely to the target area	Repeatability/Payload: 0.1 mm/5 kg
Neuronavigation	Visualization of the brain and the TMS tool based on medical imaging data and CAD data	Compatible file format: DICOM, NIfTI

## Materials and methods

### Robotic coil positioning

**Figure 2:**
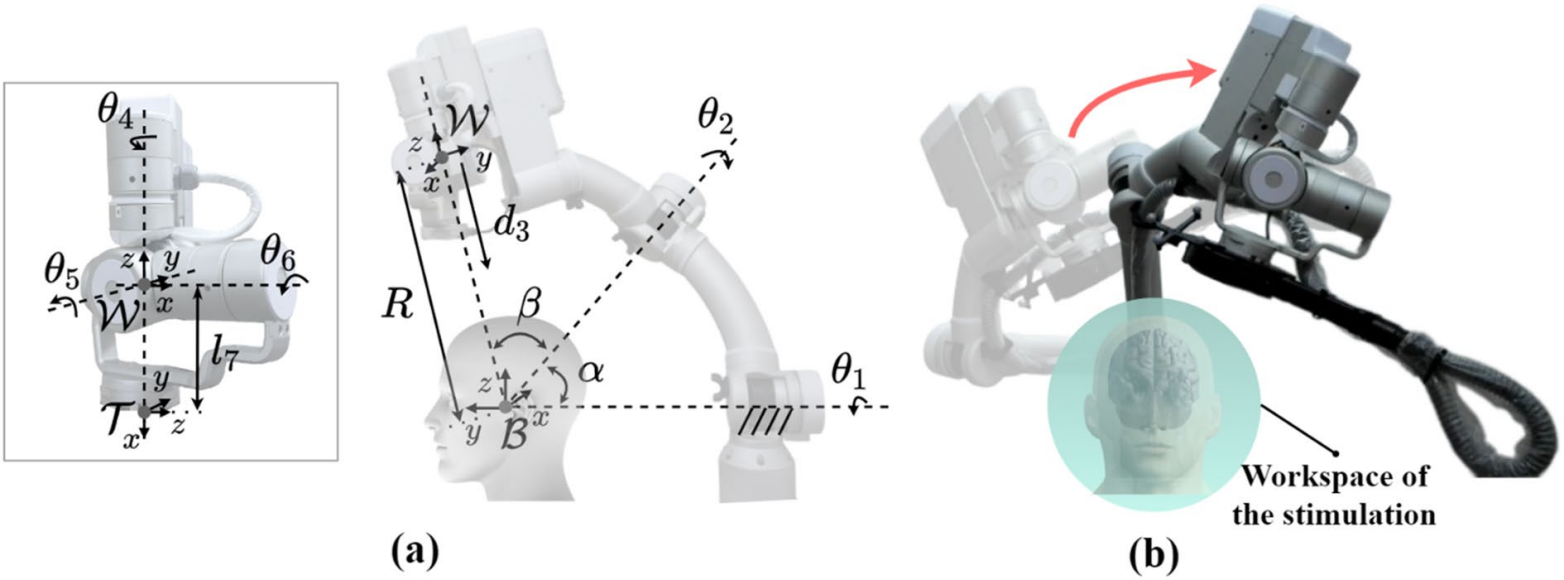
(**a**) Schematic diagram of the positioning robot, (**b**) sphere workspace over the head.

A positioning robot for noninvasive brain stimulation was developed to improve the repeatability and accuracy of stimulation^20^. Because industrial robots have a vertically articulated architecture, if they are used to stimulate the human brain, the path of a stimulator will include the area around the human head. To address this issue, we developed our initial robotic rTMS model as a serial arm with a spherical mechanism, which not only improved safety but also performed effective movements around the brain. The initial model had an hemi-spherical workspace with a radius of 350 mm, a depth of 50 mm for stimulation, and a payload of 2 kg^20^. In this study, an improved robot was developed with increased workspace and payload for stimulation of the prefrontal cortex and improved the stability of the system. Figure 2a represent the schematic diagram of the improved robotic rTMS model, $\theta_{1,2,4,5,and 6}$ and $d_{3}$ are active joint variables, whereas the link parameters $\alpha ,\beta ,R$ and $l_{7}$ are fixed variables.

The positioning robot maintains the mechanical advantages of the initial model because it is designed with equal kinematics that a spherical type as shown in Fig. 2. In addition, the improved model expand an available spherical workspace by designing with a radius of 450 mm, a depth of stimulation of 150 mm considering the average size of the human head^26^ and a payload of 5 kg in consideration of TMS coil including the cable. Furthermore, to provide an optimal workspace for various patient heights, a vertical linear stage was installed on the base, as shown in Fig. 1b. The height of the system was manually adjusted after the patient sat on a chair and was maintained during treatment. Each joint has a braking system and a force/torque (FT) sensor attached between the stimulator and the end-effector of the robot arm. Hence, the robot is stopped immediately when unwanted events are sensed by the FT sensor.

**Figure 3:**
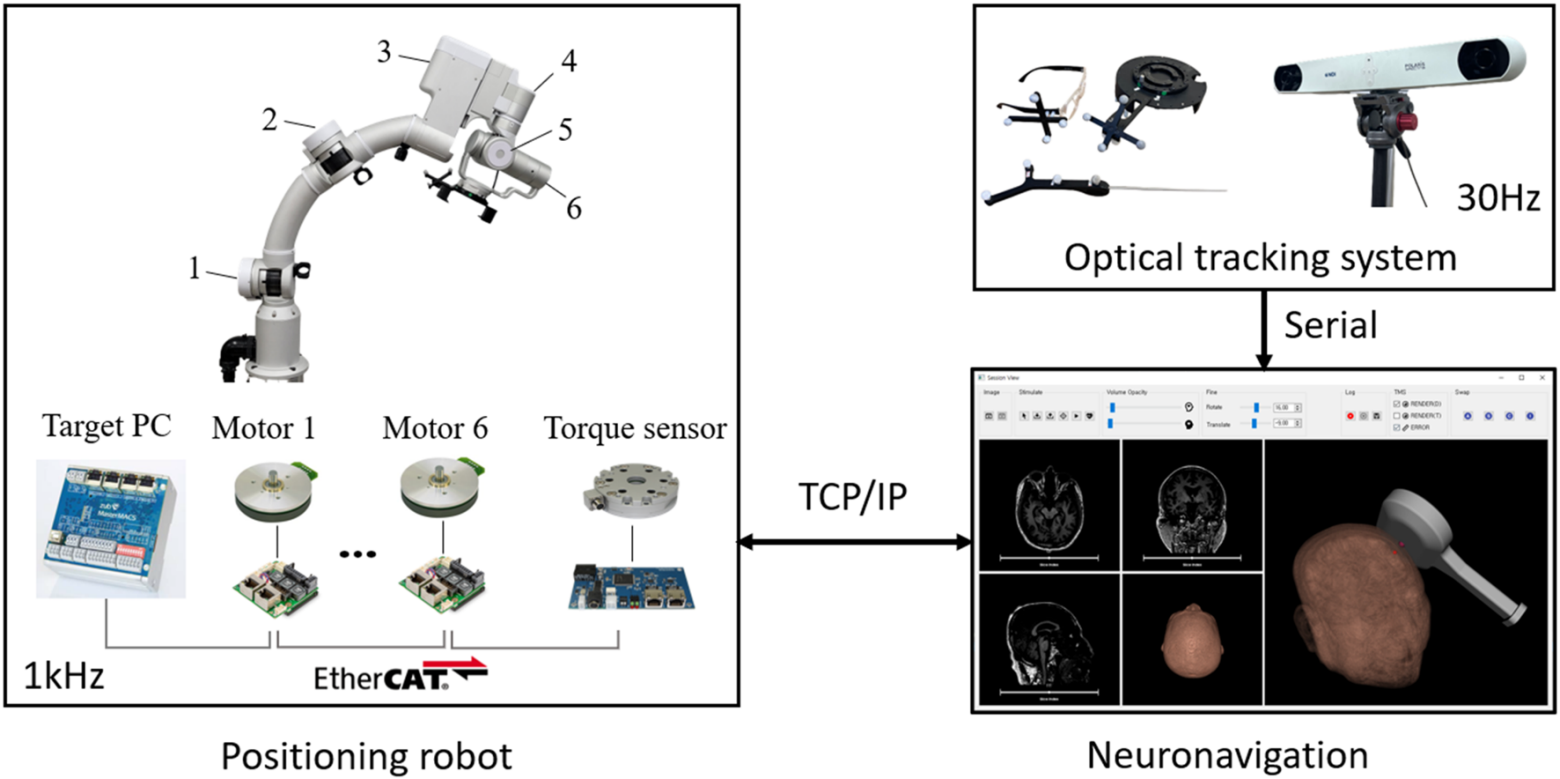
Hardware structure diagram of the positioning robot with neuronavigation.

To control the end-effector of the robot, we applied the analytical inverse kinematics solution to avoid singularities of the robot and limit the joint values^20^. For the trajectory of each joint, the robot control loop plans a minimum jerk trajectory considering the jerk limit, which is the rate of change of acceleration with respect to time, to increase safety. The positioning robot was controlled at 1 kHz, whereas the sampling rate of optical tracking system with two passive markers was 30 Hz as shown in Fig. 3.

Even if the TMS coil is properly positioned, the patient may have difficulty maintaining a fixed posture because the session time is not short; the procedure takes approximately 20 min. To compensate for this unanticipated head movement, the robot moves by estimating the movement of the patient with the tracking system. We purposely provided a latency of 500 ms to ensure patient safety, which is the time required to compensate for errors.

Coil positioning using a robot has some advantages in an rTMS treatment session: (1) the placement of the coil by the robot is more accurate than by hand; (2) it is faster and more consistent than the control by hand despite the reduced speed of the robot in consideration of the safety of the patient; (3) the repeatability of the treatment is improved because the target poses about patients are saved by neuronavigation.

### Neuronavigation

Our developed graphical user interface (GUI) including neuronavigation helps localize stimulation accurately by confirming the location of the stimulation using MRI data. Before intraoperative neuronavigation, patient registration was performed using a 3D tracking system. During the registration process, reflective markers were used to determine a patient’s spatial point precisely in a 3D space. The patient was equipped with a passive reflective marker attached to eyeglasses, which are easy to wear. Each patient was registered once.

**Figure 4:**
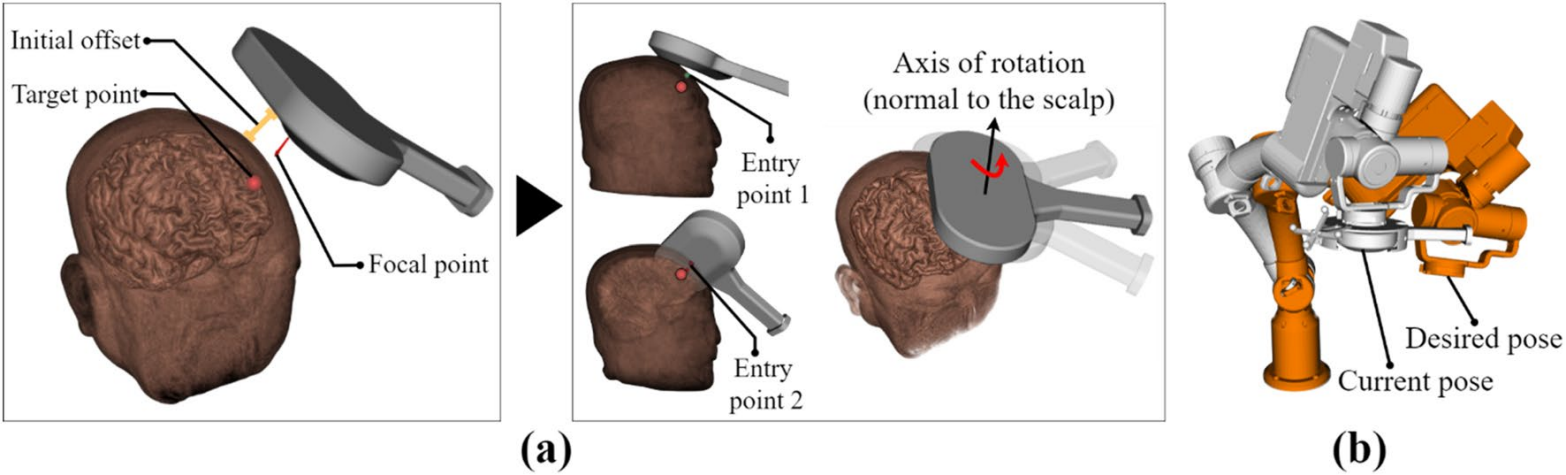
(**a**) Target pose with the initial offset and two different entry points, followed by rotation in the axis of the stimulation, (**b**) visualization of the robot movement in neuronavigation.

As the 3D model of the patient’s brain is identified using the skull-stripping method^27^, the desired cortical region can be easily selected as the target point. As the orientation of the TMS coil also affects effective stimulation^28,29^, the direction of the stimulation axis was determined after an entry point on the scalp was selected, as shown in Fig. 4a. The entry point on the scalp was selected when the reflective marker was within the field of view of the tracking system and when the contact between the TMS coil and the patient was well established. As the current and desired states of the robot were visualized in the navigation, as shown in Fig. 4b, whether the frame of the robot and the patient’s head will collide can be verified. Clinicians selected the DLPFC region for treating depressed patients based on 3D brain models created using MRI data. The selected position is sent to the robot as target pose via TCP/IP communication. Therefore, the robot could move to the registered stimulation point directly after the first treatment session. As a collision may occur on the path if the robot moves immediately to the optimal target pose, the robot is initially moved to the position with an offset and adjusted to the final target, as displayed in Fig. 4a. The axis of the stimulus was changed by selecting different entry points, although the target point remained the same. Sometimes, we needed to change the entry point if the patient felt uncomfortable or if the workspace of the robot was inaccessible with the given initial pose.

## Experiments

### Participants

Patients with major depressive disorder (MDD) according to the Diagnostic and Statistical Manual of Mental Disorders-5 (DSM-5) and aged between 20 and 80 years were recruited at Incheon St. Mary’s Hospital (Incheon, South Korea)^30^. The exclusion criteria were as follows: (1) other neurological and psychiatric disorders, including epilepsy and traumatic brain injury, (2) structural brain abnormalities on magnetic resonance imaging (MRI), (3) a history of brain surgery, (4) implanted medical devices or other metallic objects in the body, (5) pregnancy, and (6) contraindications to MRI. Fifteen patients with MDD were recruited, and one patient withdrew consent during the screening procedures. The remaining 14 patients were randomized to the robotic (n = 8) or manual (n = 6) rTMS group. One patient in the robotic rTMS group withdrew consent after the third rTMS session due to a mild and transient headache. Therefore, seven patients in the robotic rTMS group and six patients in the manual rTMS group completed the treatment sessions and underwent both the baseline and follow-up assessments.

This study was approved by the Institutional Review Board of Incheon St. Mary’s Hospital (approval number: OC20DNSI0165) and performed in accordance with the principles of the Declaration of Helsinki. Informed consent was obtained from all the participants.

### Experimental procedure

**Figure 5:**
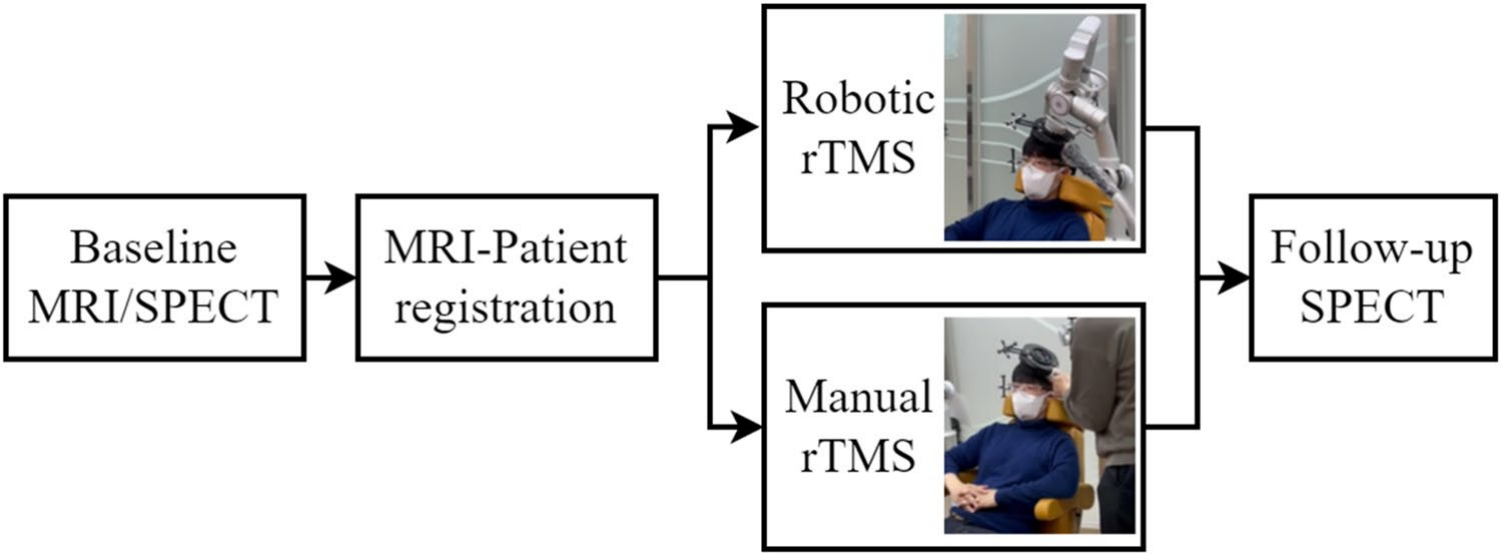
Experimental procedure. *MRI* magnetic resonance imaging, *rTMS* repetitive transcranial magnetic stimulation, *SPECT* single-photon emission computed tomography.

After the screening procedures, including electroencephalography and brain MRI, the patients were randomized to receive a total of nine sessions of either robotic or manual rTMS treatment over 3 weeks. All the patients were administered antidepressants, and the dosage was not changed during the study. Each patient was informed regarding whether they were in the group in which the positioning robot guided the coil position or the group in which a passive holder was applied. Figure 5 shows the experimental procedure for comparing the positioning robot and the traditional method for depressed patients.

Standard rTMS treatment was conducted using ALTMS (REMED, Daejeon, Korea) with a figure-of-eight-shaped coil. Before the session, the motor threshold (MT) was estimated by stimulating the primary motor cortex and detecting the minimum level of stimulation energy required to induce contraction of the contralateral abductor pollicis brevis muscle. For the group undergoing robotic rTMS, the clinician first determined the cortical entry point where the coil was in contact with the scalp. Unlike the target cortical region, we identified two or three possible entry points. If the contact between the TMS device and the patient caused discomfort, the entry point was excluded. After the first session, the robotic coil was automatically moved to the entry point, without the help of the clinician.

**Figure 6:**
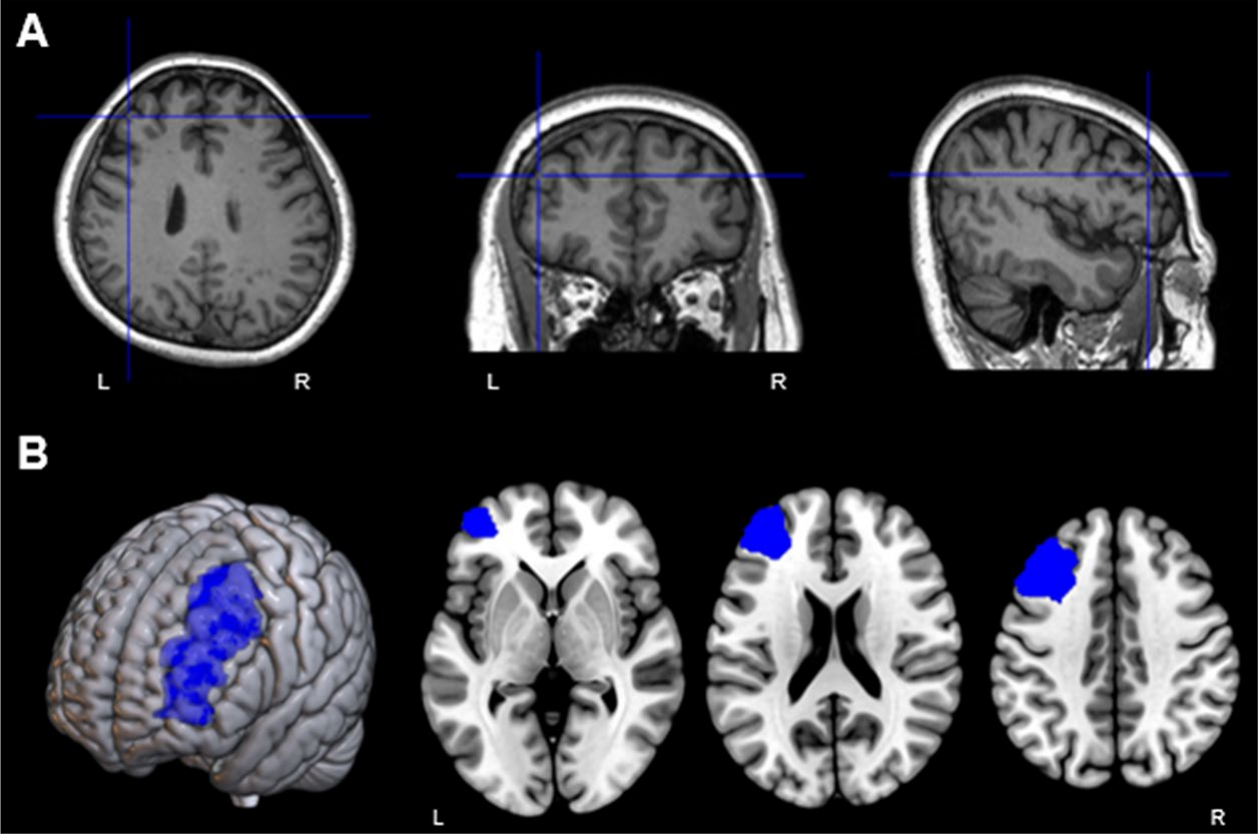
(**a**) Target brain region for the robotic repetitive transcranial magnetic stimulation (rTMS) treatment of a patient. (**b**) Region of interest for single-photon emission computed tomography (SPECT) analysis.

Table 2 summarizes the applied parameters for treating depression. The stimulation intensity was 100% of the individual MT. As shown in Fig. 6a, the site of the stimulation was over the left DLPFC. As summarized in Table 2, the frequency of stimulation was set to 20 Hz for 2 s^31–33^, with an intertrain interval of 28 s. In comparison to 1 Hz, 10 Hz, or intermittent theta-burst stimulation, 20 Hz stimulation may have more consistent excitatory effects, according to neurophysiological and functional magnetic resonance imaging (fMRI) findings^34^. Each treatment session lasted for 20 min.

**Table 2:** Parameters for repetitive transcranial magnetic stimulation (rTMS) treatment.

Parameter names	Parameter values
Treatment coil location	Left DLPFC
Number of pulses per session	1600
Magnetic field intensity	100% of MT
Pulse repetition rate	20 pulses per second
Stimulation time	2 s
Stimulation interval	28 s
Length of treatment session	20 min

*DLPFC* dorsolateral prefrontal cortex, *MT* motor threshold.

During the baseline and follow-up visits, the patients underwent brain SPECT scans and completed the Beck Depression Inventory (BDI-II)^35^, which consists of 21 items on a four-point scale from 0 to 3. The maximum total score of the BDI-II was 63, with higher scores indicating greater severity.

### Brain imaging and analysis

Brain MRI images were acquired using a 3T Siemens Skyra scanner (Siemens, Erlangen, Germany) with a 32-channel head coil. The following scans were performed: T1-weighted magnetization-prepared rapid gradient echo (TR = 2,000 ms, TE = 2.49 ms, FOV = 230 × 230 mm^2^, matrix = 256 × 256, flip angle = 9°, voxel size = 0.9 × 0.9 × 0.9 mm^3^) and fluid-attenuated inversion recovery (TR = 9,000 ms, TE = 81 ms, inversion time = 2500 ms, FOV = 224 × 224 mm^2^, matrix = 256 × 320, flip angle = 90°, voxel size = 0.7 × 0.7 × 4.0 mm^3^).

Brain SPECT data were obtained using a dual-head gamma camera (Discovery NM630; GE Healthcare, Milwaukee, WI, USA) equipped with a low-energy fan-beam collimator. First, 555–740 MBq of technetium-99 m hexamethylpropylene amine oxime (99mTc-HMPAO) was intravenously injected in all patients before the SPECT scan was performed. All the patients rested for 40 min until the acquisition started. During image acquisition, the patients were in a supine position, and the camera was rotated for a total of 720°, with an interval of 6°, at a rate of 12 s per frame. The continuous transaxial images were reconstructed with the following parameters: voxel size = 1.95 × 1.95 × 2.08 mm^3^, matrix = 128 × 128 mm^2^, field of view = 250 × 250 mm^2^, and 20% symmetric energy window at 140 keV. The standard ordered subset expectation maximization algorithm (six iterations and 10 subsets) was applied with a Butterworth filter (cut-off frequency = 0.5 cycles/pixel, power = 10).

All the SPECT images were preprocessed using Statistical Parametric Mapping 12 (SPM). The images were nonlinearly registered to the standard SPECT template and resliced with a voxel size of 2.0 × 2.0 × 2.0 mm^3^. The global rCBF was scaled to 50 ml/dl/min, and proportional scaling was applied for normalization. The region of interest (ROI) for the left DLPFC was defined as the left middle frontal gyrus in the automated anatomical labeling atlas^36^ as shown in Fig. 6b. For each patient, normalized rCBF values in the ROI were extracted using the MarsBar toolbox^37^ to calculate the contrast effect before and after rTMS.

### Statistical analysis

Baseline differences in demographic and clinical characteristics were assessed between the robotic and manual rTMS groups using the Mann–Whitney U test or Fisher’s exact test. The changes in BDI-II scores and rCBF in the left DLPFC ROI were compared between the two groups using the Mann–Whitney U test. For each group, the changes in these two measures were evaluated using the Wilcoxon signed-rank test. A two-tailed *p* value of < 0.05 was considered significant. Statistical tests were conducted using STATA version 17 (StataCorp., College Station, TX, USA).

## Results

### Position error analysis

We recorded the pose of the head using a 3D measuring device to determine the extent to which head movement occurred during the rTMS session. The subjects were instructed to minimize head movement during the rTMS treatment. As the patient’s neck was not fixed, the error tended to increase gradually over time.

We designed two experiments to compare the accuracy of the manual adjustment method with that of the robot-assisted method. The first experiment was conducted to determine how precisely and quickly the focus of the coil reaches the target region of the cortical area. The second experiment measured the extent to which the error changes during the session. In the first experiment, the time required for the coil to align with the target was measured. The manual adjustment method required approximately twice as long as the robotic method, as shown in Table 3. This time difference is already large. However, this difference could be more prominent if we consider two experimental conditions. First, manual adjustment stops when the clinician feels that he or she cannot adjust the coil better than the current error, which is usually considerably larger than the error from robotic adjustment. Second, the speed of the robot was reduced along the path, including some waypoints, for patient safety. In this study, the time taken for the registration process was not considered.

**Table 3:** Preparation time and accuracy of the initial coil placement.

Method	Coil placement time (s)	Position error (mm)	Orientation error (deg)
Robotic	16.10 ± 4.62	0.94 ± 0.30	0.11 ± 0.23
Manual	34.50 ± 12.46	12.49 ± 5.10	6.24 ± 1.08

The manual method has a large error compared with the robotic method, as shown in Table 3. As the position and orientation of the coil must be adjusted simultaneously manually, controlling the coil is difficult. In practice, an additional error inevitably occurs when the joints in the fixing arm are locked to maintain the desired pose. Table 3 indicates that the mean position and orientation errors with the robotic and manual methods are 0.94 mm and 12.49 mm, and 0.11° and 6.24°, respectively.

The time graph of the initial coil placement in all groups was depicted as shown in Figs. S1 and S2 (in Supplementary Information). In our previous study on the placement experiment for the focused ultrasound stimulation^21^, the position and orientation errors were 7.1 mm and 6.0°, respectively. As the ultrasound transducer weighs less than the TMS coil, it is more comfortable to align the target position manually. Therefore, the accuracy of the placement was higher than that of our experiment.

**Figure 7:**
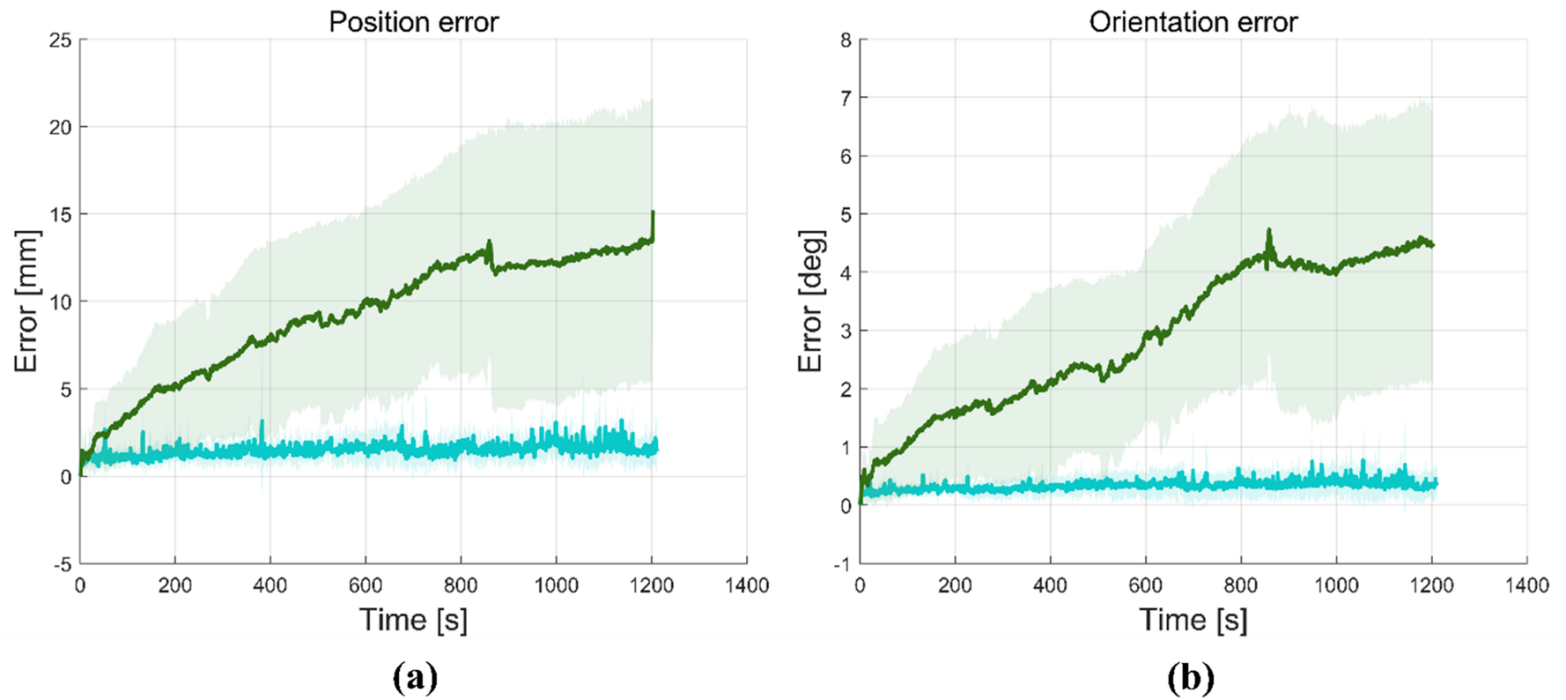
Mean amplitude of head motion over time for the two methods: the robotic rTMS with head motion compensation (cyan) and the manual method (dark green), (**a**) position error, (**b**) orientation error.

As shown in Table 4, the changes in the position and orientation using the two methods are summarized for four time points with the mean values for all the measurement scenarios (see Fig. S3 in Supplementary Information for details). Each error refers to an additional displacement from the initial position of the coil. Therefore, although some of the errors in Table 4 are smaller than those in Table 3, the measurements shown in Table 4 for head movement during the treatment session excluded the initial error. Figure 7 indicates that the error in the manual method (dark green) increased to 66% of the final position error within 5 min. After 10 min, the position was changed by 9.81 mm from the initial placement for the manual method. Using motion compensation with the robot, the final position and orientation (cyan) were 1.43 mm and 0.32°, respectively.

**Table 4:** Position and orientation errors during the repetitive transcranial magnetic stimulation (rTMS) session.

	5 min	10 min	15 min	20 min	Mean
Position error					
Robotic	1.70 ± 0.63	1.43 ± 0.99	1.47 ± 0.80	1.11 ± 0.76	1.43
Manual	7.40 ± 2.68	9.81 ± 2.86	10.21 ± 3.74	11.17 ± 4.00	9.65
Orientation error					
Robotic	0.30 ± 0.16	0.33 ± 0.22	0.37 ± 0.22	0.28 ± 0.23	0.32
Manual	2.11 ± 1.35	3.05 ± 1.84	3.65 ± 2.28	4.06 ± 2.54	3.22

### Clinical and neuroimaging results

The baseline demographic and clinical characteristics are summarized in Table 5. There were no significant differences in age (z = 1.1, *p* = 0.27), sex (*p* = 1.00), or BDI-II (z = − 0.13, *p* = 0.90). After the rTMS treatment, the robotic rTMS group showed a trend-level decrease in the BDI scores (z = − 1.95, *p* = 0.05), whereas the manual rTMS group did not (z = − 1.48, *p* = 0.14). However, the changes in the BDI scores did not significantly differ between the two groups (z = − 0.36, *p* = 0.72) as shown in Table 6.

**Table 5:** Baseline characteristics of the study participants.

Characteristics	Robotic rTMS (n = 8)	Manual rTMS (n = 6)	*p*
Age	71.9 ± 9.5	68.0 ± 6.6	*p* = 0.27
Sex (male/female)	2/6	1/5	*p* = 1.00
BDI-II	24.6 ± 13.6	25.0 ± 8.0	*p* = 0.90

*BDI-II* Beck Depression Inventory-II, *rTMS* repetitive transcranial magnetic stimulation.

**Table 6:** Changes in clinical and neuroimaging outcomes after rTMS treatment.

Outcomes	Robotic rTMS (n = 7)		Manual rTMS (n = 6)		*p* (between-group)
	Value	*p* (within-group)	Value	*p* (within-group)	
BDI-II	23.1 ± 14.0 to 19.7 ± 14.9	0.05	25.0 ± 8.0 to 22.3 ± 8.0	0.14	0.72
rCBF in the left DLPFC	76.6 ± 2.5 to 79.3 ± 3.2	0.06	78.8 ± 3.0 to 79.9 ± 4.4	0.60	0.25

*BDI-II* Beck Depression Inventory-II, *DLPFC* dorsolateral prefrontal cortex, *rCBF* regional cerebral blood flow, *rTMS* repetitive transcranial magnetic stimulation.

SPECT analysis showed a marginal significance in the rCBF increases in the left DLPFC in the robotic rTMS group (z = 1.86, *p* = 0.06), but not in the manual rTMS group (z = 0.52, *p* = 0.60). Between-group differences in the rCBF changes in the left DLPFC were not significant (z = − 1.14, *p* = 0.25) as shown in Table 6.

### Questionnaire

Although a robot can be used to increase accuracy and select a location quickly, side effects may occur when applied to a patient. A questionnaire was created to investigate these effects. It consists of five items inquiring about patients’ personal feelings after the robotic rTMS treatment. The five items are related to comfort with robotic rTMS (1-very uncomfortable to 5-very comfortable), setup time (1-feeling too long to 5-acceptable setup time), the repeatability of rTMS positioning during multiple sessions (1-very different to 5-very consistent), the intensity of rTMS during multiple sessions (1-quite different to 5-very consistent), and the safety of robotic rTMS (1-feeling very unsafe to 5-feeling very safe).

**Figure 8:**
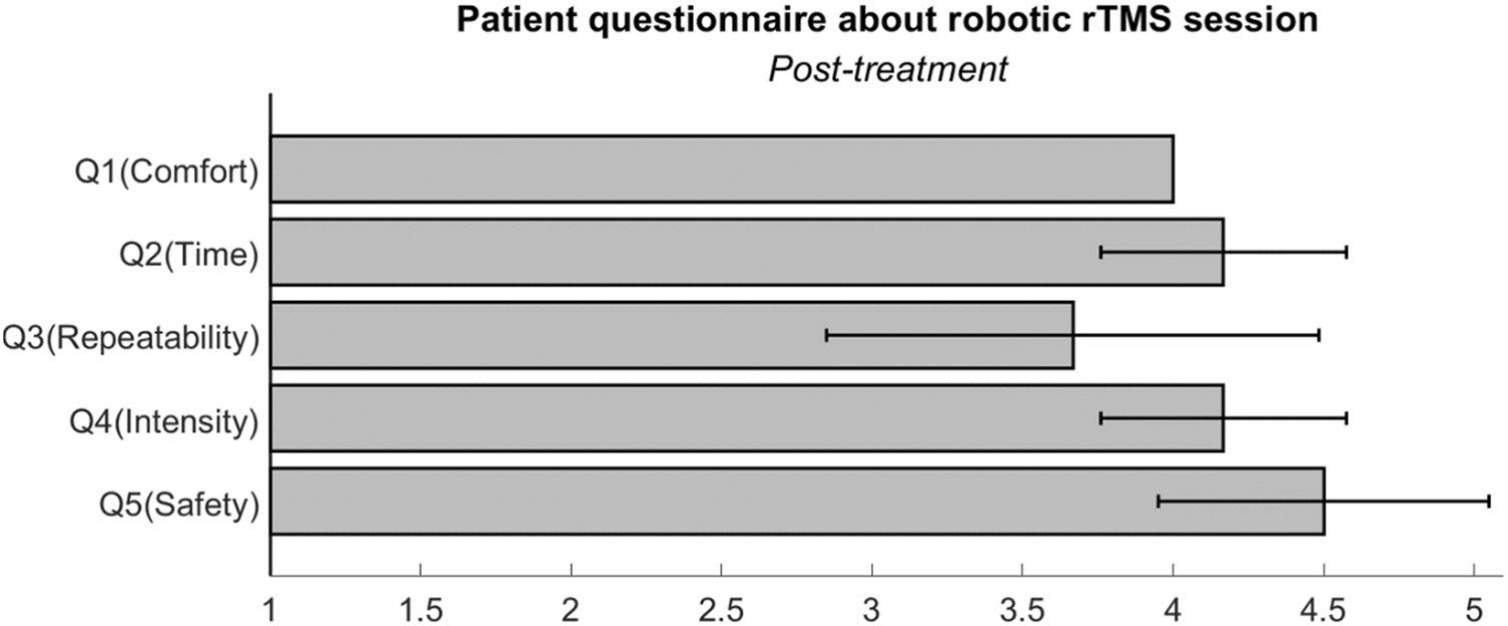
Patient questionnaire about the robotic repetitive transcranial magnetic stimulation (rTMS) session.

Notably, the average score for safety (Q5) was high as shown in Fig. 8. Although the coil was positioned using a robotic device without the help of a clinician, the patients did not feel anxious or afraid. In practice, as the major limitation of robotic rTMS is patient safety, this result provides evidence for the feasibility of the robot-assisted system. All the patients provided a score of 4 for comfort (Q1).

As expected, the patients felt that the coil placement time was appropriate as indicated in the time (Q2) response. This finding is directly related to our analysis as shown in Table 3. Surprisingly different from our expectations, a few patients felt that the coil was not always exactly positioned in the same region as indicated in the repeatability (Q3) response. Our autonomous system monitors and maintains the position error within a given small value. Therefore, there could be some explanations on this. It may be just personal feelings or one possibility is that our wearable marker may have been slightly moved by the patients during the session. We asked the patients not to touch the marker on their head during the session, but it was noticed that some patients unconsciously touched it. A slight deviation of the reflective marker on the head directly leads to a relative position error between the coil and the patient, resulting in different stimulation points in the brain. Therefore, we need to develop a better wearable marker with improved wearability and immobility in the future, or at least a detection algorithm to detect this kind of unintentional change. Moreover, some patients felt stimulated at different intensities in each session, as shown in the intensity (Q4) response. The main reason for this is believed to be what it called “sensory adaptation” to the stimulus during the session. As shown in Tables 3 and 4, the target position of the robotic rTMS is accurate, even though the patients felt that they were stimulated in inconsistent regions.

## Conclusion

During rTMS treatment, the stimulation location should not be changed for a long time, and it is tedious and time-consuming to locate the same stimulation location in every session. Previous studies focused on developing the robotic coil placement system and validating its accuracy with subjects. In this study, we recruited 15 patients with depression (including dropouts) and compared the effects of rTMS treatment using the robotic and manual methods through two analyses. First, we evaluated the setup time and accuracy through coil placement experiments of two methods. Second, SPECT and BDI-II were measured before and after rTMS to compare the therapeutic effects on depression. In a parallel study, the patients were randomized into two groups that received either manual or robotic rTMS treatment only.

The preparation time was reduced by 53% when using the robot compared with that using manual treatment. The initial placement by the robot had the position error of 0.94 mm and the orientation error of 0.11° on average. In the manual method, the corresponding errors were 11.17 mm and 4.06°.

The results from the clinical and neuroimaging assessments indicated comparable improvements in depression severity and rCBF in the left DLPFC between the robotic and manual rTMS groups. However, as the statistical tests did not reach significance, the clinical superiority of the robotic rTMS treatment cannot be proven from our preliminary results. Due to the small sample size in this pilot study, further larger studies should be conducted to evaluate the clinical advantages of the robot-assisted rTMS system using various assessment tools. In addition, potential confounding effects such as antidepressant type and dosage should be considered in future studies. Although the accuracy and precision of coil placement were relatively different between the two groups, the results of the clinical inspection indicated that there was no significant difference. This might be because cortical tissue, which can affect treatment for depression, occupies a large area rather than because of a variation in the accuracy for the different stimulation methods^38–40^. Therefore, future research is necessary to validate the conclusions drawn from this study.

We additionally administered a simple questionnaire that inquired about the safety of the robot and the preparation time for the group to which the robot was applied. Interestingly, the response for safety was rated the highest, confirming that the patients felt that robotic rTMS was a safe and reliable method. We believe that the safe rounded design and soft movement of our rTMS robot contributed to this positive evaluation.

### Supplementary Information

Supplementary Figures.

## Data Availability

The datasets analyzed during the current study are not publicly available because the IRB has restrictions on sharing datasets. Requests to access the datasets should be directed to Yong-An Chung (yongan@catholic.ac.kr).
