# Supplementary material for: Robotic transcranial magnetic stimulation in the treatment of depression: a pilot study
Source: Sci Rep. 2023 Aug 28;13:14074. doi: 10.1038/s41598-023-41044-1 (PMC10462606; doi:10.1038/s41598-023-41044-1)
Supplement: Supplementary file 1 — Supplementary Figures. [file 41598_2023_41044_MOESM1_ESM.docx]

**Supplementary Information**

**Robotic transcranial magnetic stimulation in the treatment of depression: a pilot study**

Hyunsoo Shin^1,†^, Hyeonseok Jeong^2,3,†^, Wooseok Ryu^4^, Geunhu Lee^1^, Jaeho Lee^1^, Doyu Kim^5^, In-Uk Song^3^, Yong-An Chung^3,5,^* , Sungon Lee^1,^*

^1^ Department of Electrical and Electronic Engineering, Hanyang University, Ansan 15588, Republic of Korea

^2^ Department of Radiology, Incheon St. Mary's Hospital, College of Medicine, The Catholic University of Korea, Seoul 21431, Republic of Korea

^3^ Department of Neurology, Incheon St. Mary's Hospital, College of Medicine, The Catholic University of Korea, Seoul 21431, Republic of Korea

^4^ Tesollo Inc., Gwangmyeong 14353, Republic of Korea

^5^ Department of Nuclear Medicine, Incheon St. Mary's Hospital, College of Medicine, The Catholic University of Korea, Seoul 21431, Republic of Korea

* Corresponding authors: Sungon Lee (sungon@hanyang.ac.kr); Yong-An Chung (yongan@catholic.ac.kr).

^†^ These authors contributed equally to this work.


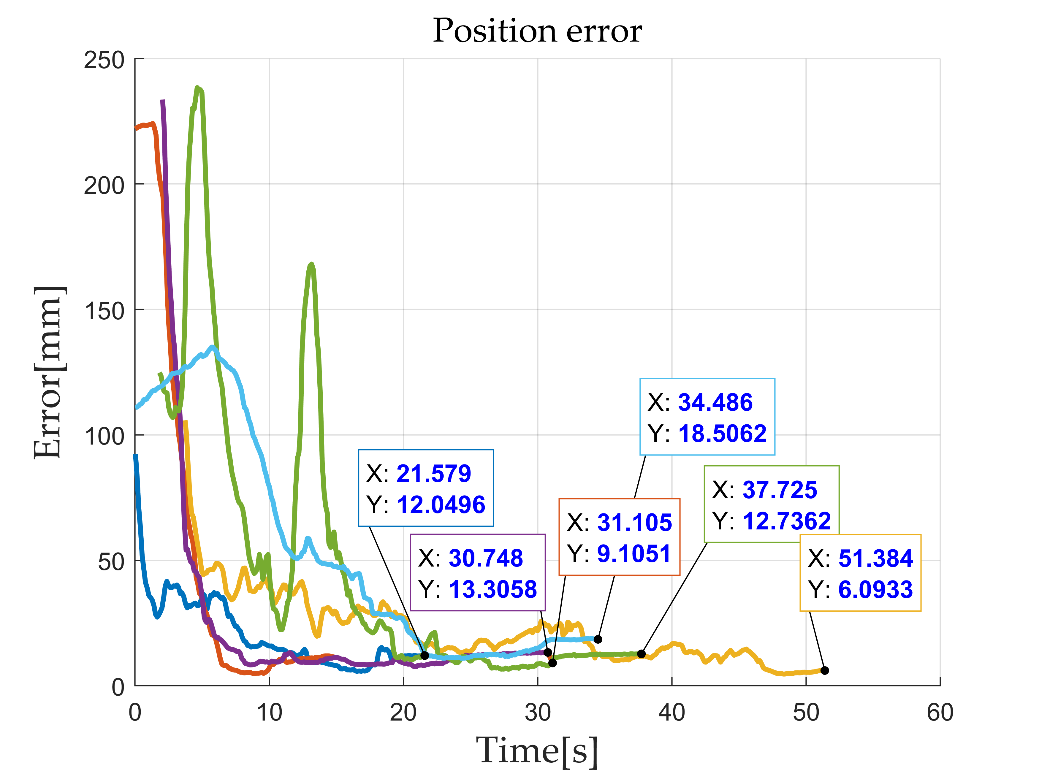


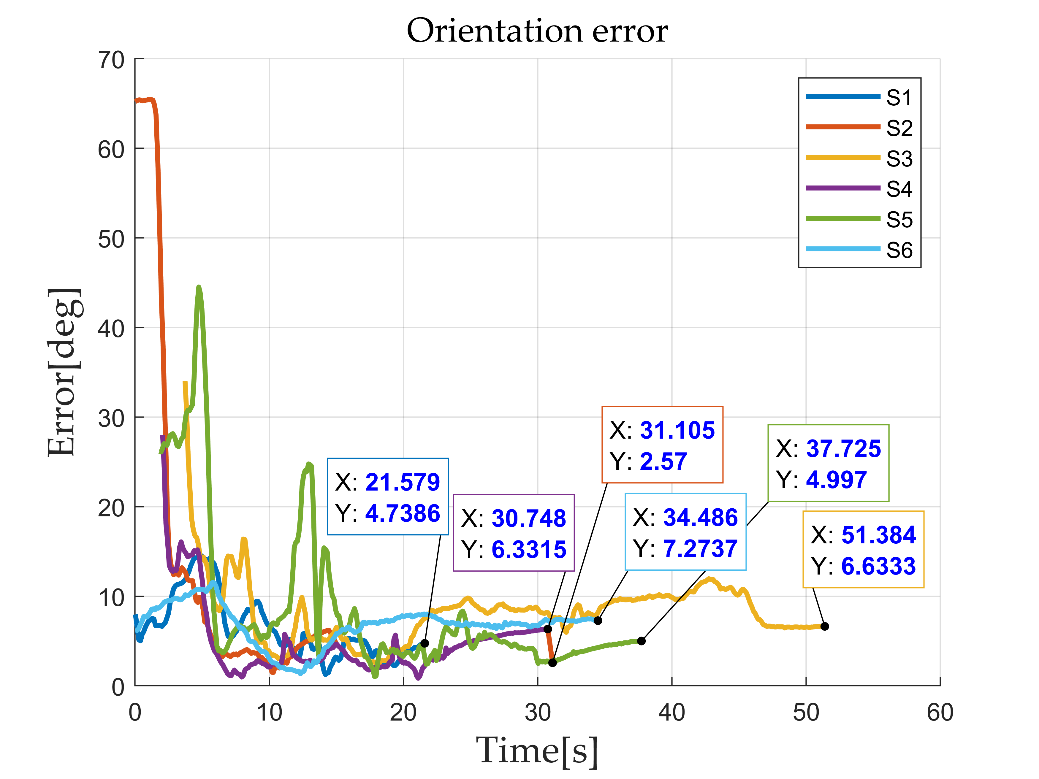


Figure S1. Coil placement time with manual method


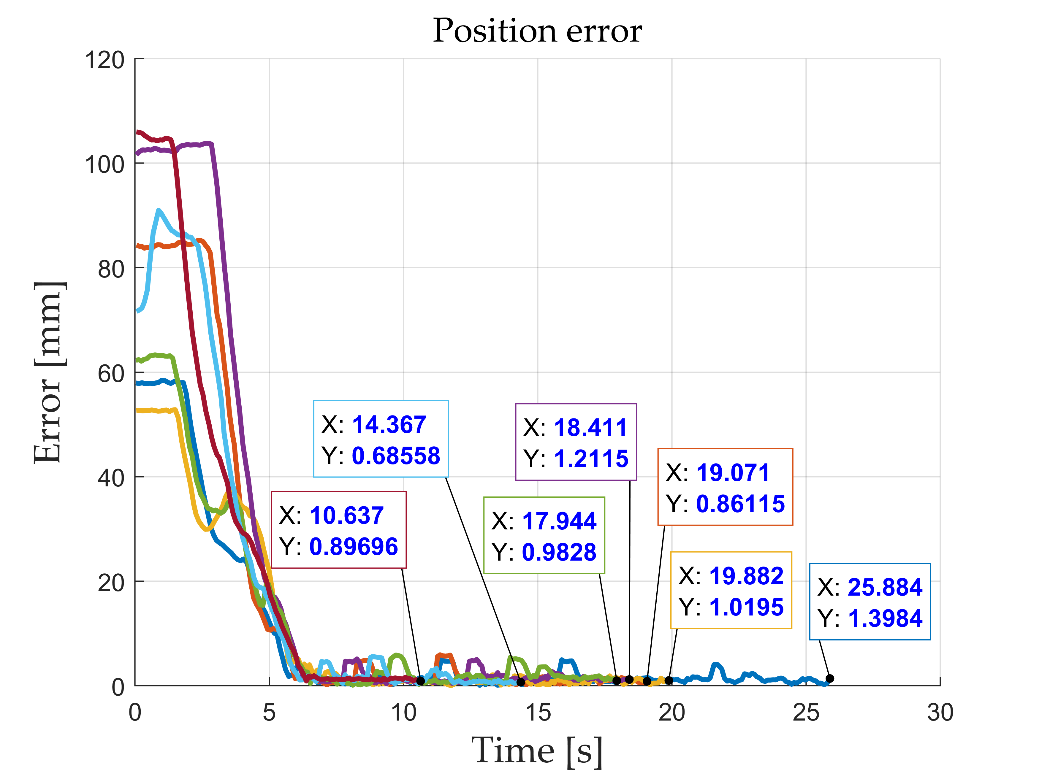

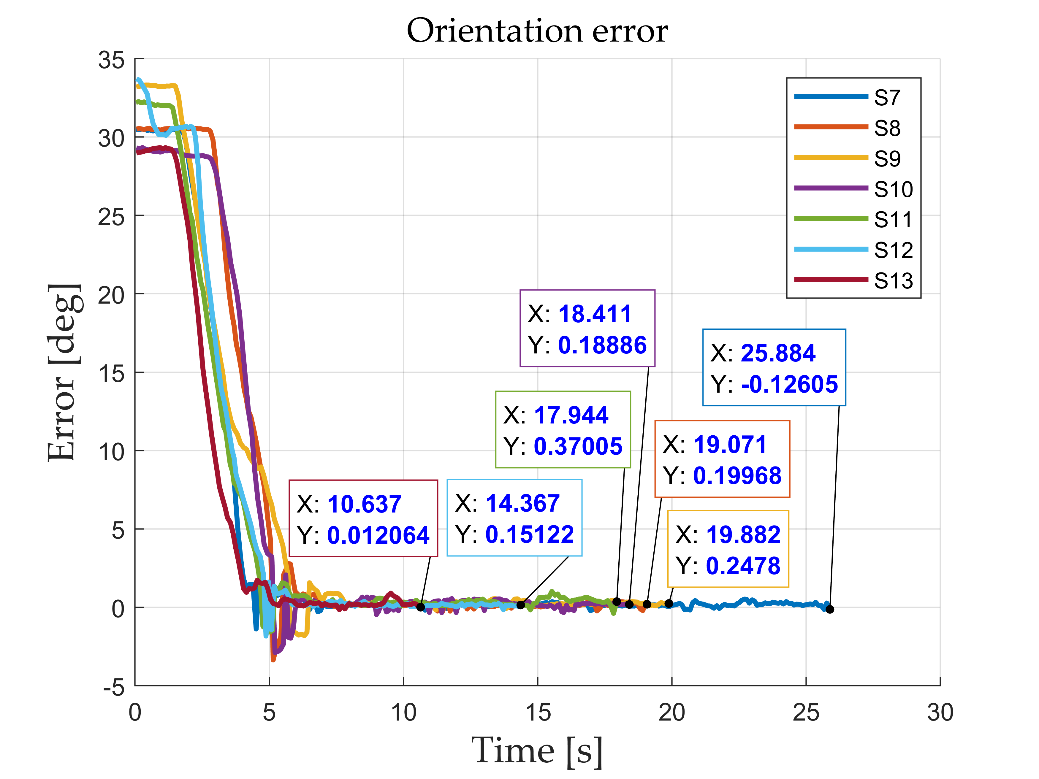


Figure S2. Coil placement time with robotic method


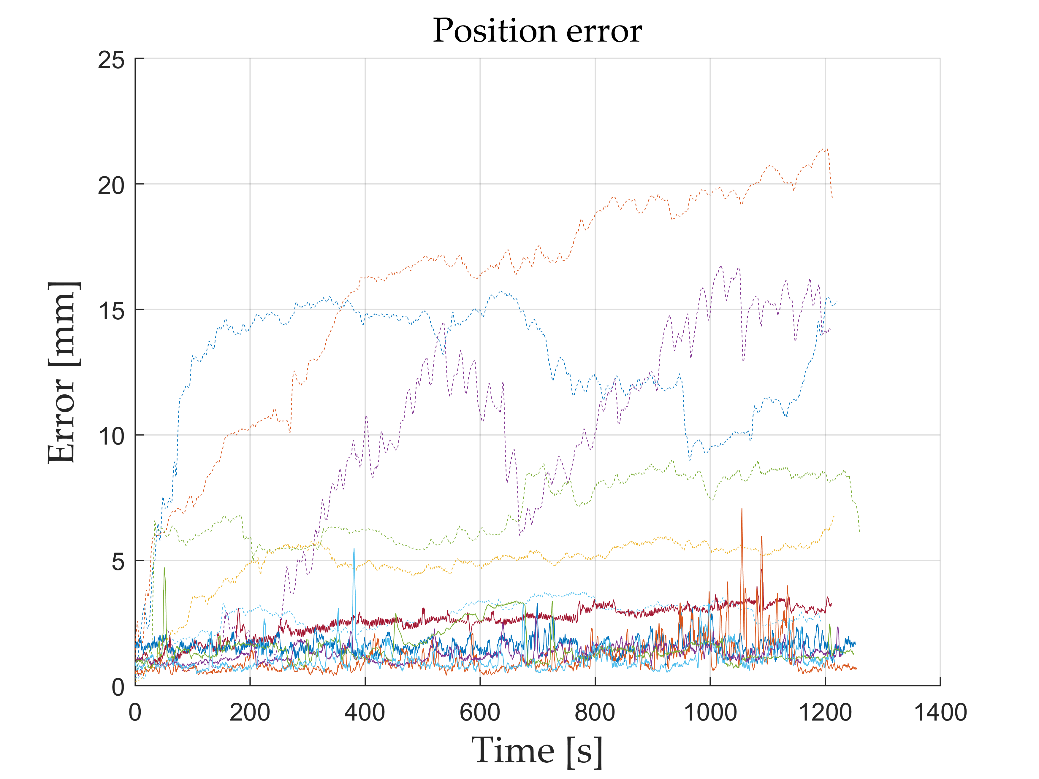


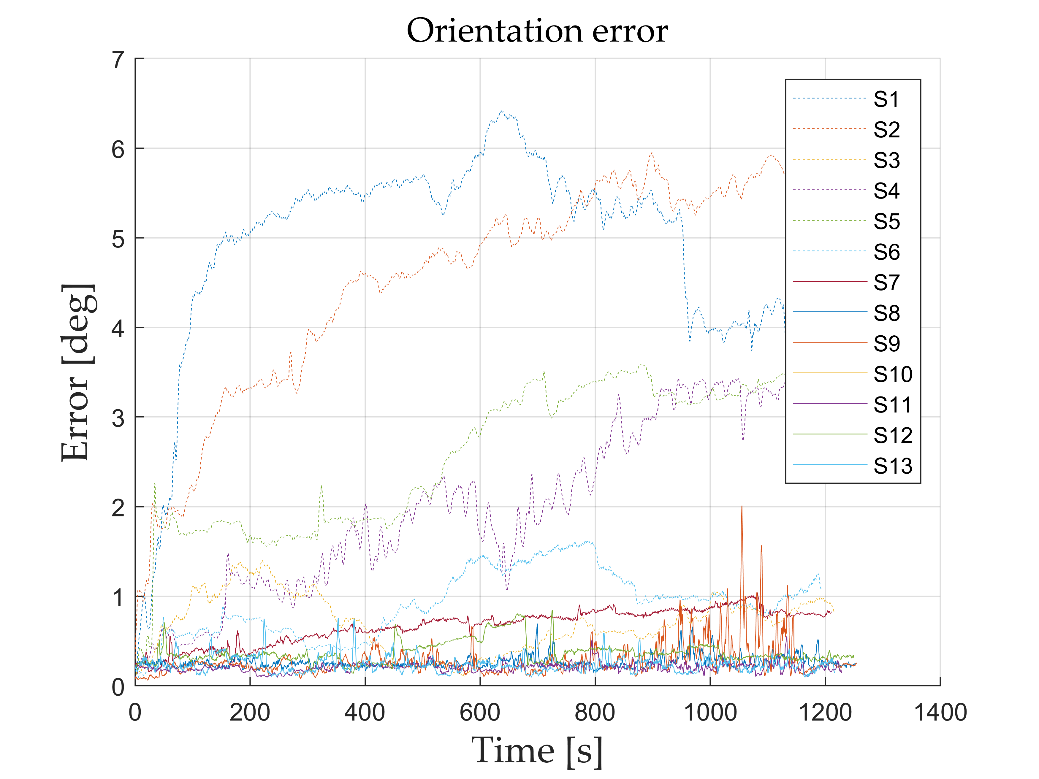


Figure S3. Position and orientation error during the treatment session
